# Quantifying nutrient recovery efficiency and loss from compost-based urban agriculture

**DOI:** 10.1371/journal.pone.0230996

**Published:** 2020-04-03

**Authors:** Paliza Shrestha, Gaston E. Small, Adam Kay

**Affiliations:** 1 Department of Biology, University of Saint Thomas, Saint Paul, Minnesota, United States of America; 2 Department of Ecology, Evolution, and Behavior, University of Minnesota, Saint Paul, Minnesota, United States of America; Trent University, CANADA

## Abstract

The use of compost in urban agriculture offers an opportunity to increase nutrient recycling in urban ecosystems, but recent studies have shown that compost application often results in phosphorus (P) being applied far in excess of crop nutrient demand, creating the potential for P loss through leachate and runoff. Management goals such as maximizing crop yields or maximizing the mass of nutrients recycled from compost may inadvertently result in P loss, creating a potential ecosystem disservice. Here, we report the results from the first two years of an experimental study in which four different crops grown in raised-bed garden plots with high background P and organic matter received one of two types of compost (municipal compost made from urban organics waste, or manure-based compost) at two different levels (applied based on crop N or P demand), while additional treatments received synthetic N and P fertilizer or no soil amendments. Because of the low N:P ratio of compost relative to crop nutrient uptake, compost application based on crop N demand resulted in overapplication of P. Crop yield did not differ among treatments receiving compost inputs, and the mass of P recovered in crops relative to P inputs decreased for treatments with higher compost application rates. Treatments receiving compost targeted to crop N demand had P leachate rates approximately twice as high as other treatments. These results highlight tradeoffs inherent in recycling nutrients through UA, but they also show that targeted compost application rates have the capacity to maintain crop yields while minimizing nutrient loss. UA has the potential to help close the urban nutrient loop, but if UA is to be scaled up in order to maximize potential social, economic, and environmental benefits, it is especially important to carefully manage nutrients to avoid ecosystem disservices from nutrient pollution.

## Introduction

Cities are hotspots of biogeochemical transformations, relying on regional and global hinterlands to supply food, water, and other materials, and to assimilate wastes [1,2]. Because human population is increasingly concentrated in urban areas [3], the importance of cities as a driver of global biogeochemical fluxes will continue to increase in coming decades [4]. As such, the potential for moving cities towards a circular metabolism, in which waste products are used as a resource, is seen as an important opportunity for advancing sustainability [5,6].

One important pathway by which urban nutrient recycling could potentially be increased is through recycling of organic waste (i.e., composting) coupled with urban agriculture (UA, defined here as any food production occurring in urban areas, whether commercial or noncommercial). Urban food production is rapidly increasing in the United States through the development of community gardens and small commercial operations [7–9], and has the potential to supply a significant fraction of local fruit and vegetable demand in some cities [10]. Many cities are expanding composting in order to keep organic waste out of landfills, and because outdoor, soil-based UA relies heavily on compost-derived nutrients [6,11,12], UA coupled with composting has the potential to recycle nutrients such as phosphorus (P) and nitrogen (N) from urban organic waste back into the human food system.

Previous analyses have identified the opportunities for recycling urban nutrients through the use of compost in urban agriculture [6,11], but evaluation of the efficiency of nutrient recycling in urban agriculture is needed to assess the sustainability of this approach. Compost-derived nutrients could be considered “recycled” as soon as they are applied to gardens, but recycling would be more appropriately quantified (and conceptualized) as the fraction of these nutrients that are recovered by crops. While nutrient use efficiency has been a focus of research in rural agriculture [13,14], it has received little attention in UA. Recent observational studies of UA have found evidence of excessive application of compost-derived nutrients leading to build-up of P (and, to a lesser degree, N) in garden soils [15–17]. Overapplication of P likely results from the relatively low N:P ratio of many composts relative to requirements for crop growth (so that applying compost based on plant N demand results in excessive P inputs) [18], as well as the general lack of economic or regulatory disincentives against applying excess nutrients in small-scale urban agriculture [19], and a “more is better” mentality by many urban gardeners result in excessive nutrient application [17,20].

Although soils have the capacity to retain P through sorption and other chemical processes, soils that accumulate P in high concentrations can export dissolved P through leachate and surface runoff [21]. Compost application rates typical of UA practitioners can result in high loss of P as leachate [22] and buildup of P in native soil below urban gardens [23]. Under UA intensification scenarios [24,25], P loss from urban agriculture could become a source of nutrient pollution to receiving waters downstream, threatening aquatic ecosystem health and undermining other ecosystem services provided by UA.

Sustainable use of compost in UA may pose a tradeoff among management goals such as maximizing crop yield, maximizing the mass of nutrients recycled, and minimizing the loss of nutrients to the environment. Here, we present the results of the first two years of a multi-year experimental manipulation aimed at understanding these potential tradeoffs and identifying best management practices. In this study, garden plots with high background soil P (typical of urban gardens [17]) received annual inputs of either municipal compost (from urban organics waste) or manure-based compost (commonly used by local UA practitioners), applied based on either the anticipated P or N demand of crops, synthetic fertilizer targeted to match crop P and N demand, or control plots which receive no soil amendments. The highest P input rate used in this study is well below the median compost P input rate that we previously documented for the local metropolitan area [17]. We hypothesize that different treatments will optimize these different metrics, indicative of tradeoffs among metrics of sustainability [22].

## Material and methods

### Site description

**Fig 1:**
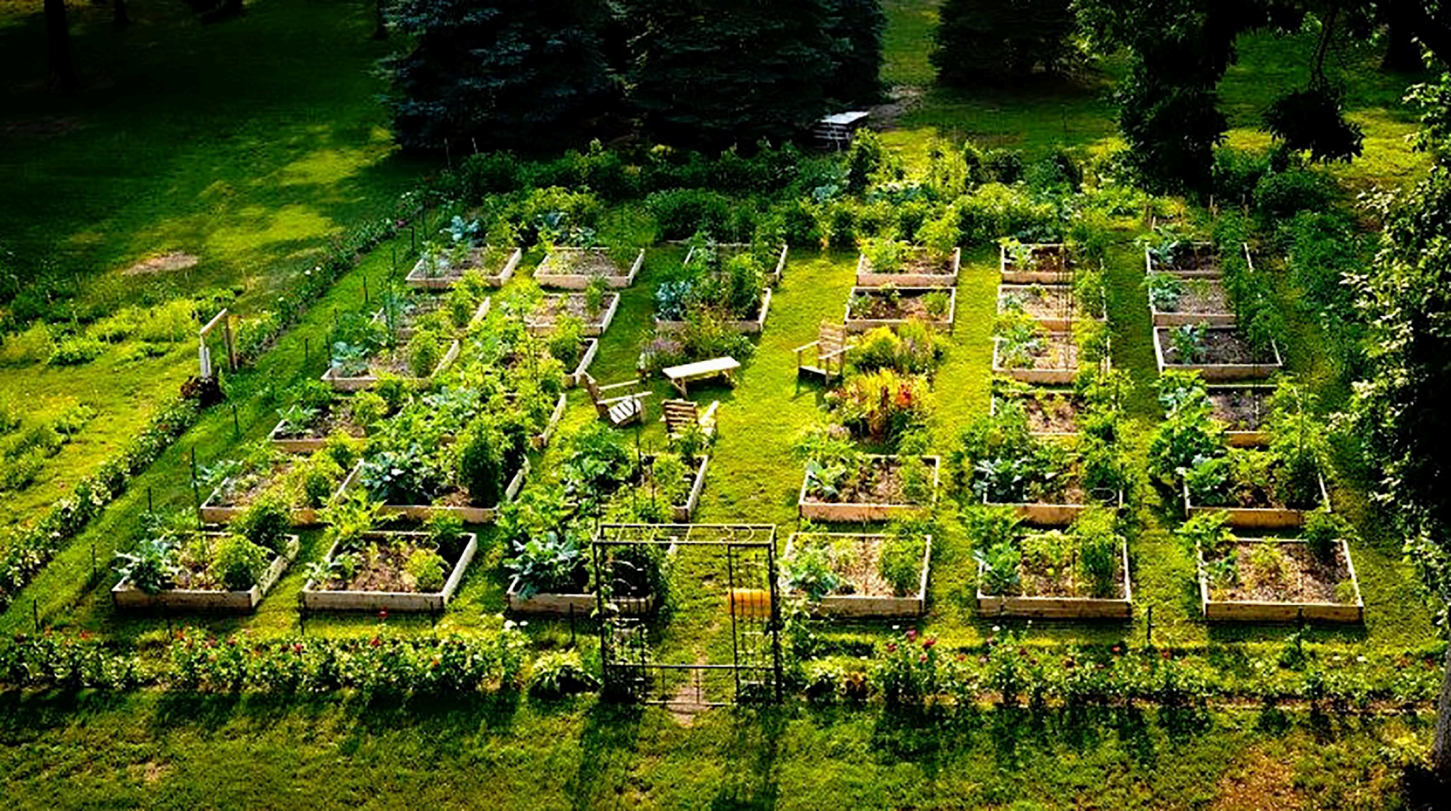
Experimental layout of the garden plots at the University of Saint Thomas campus.

The study site, located at the University of Saint Thomas (UST) Stewardship Garden, in Saint Paul, MN (N 44.938º, W 93.196 º), was originally established in 2011 and consists of thirty-two, 0.4 m deep, 4 m^2^ raised bed plots (Fig 1). From 2011–2016, individual plots had received annual inputs of 0–6 kg/m^2^ of composted cow manure, or 0–9 kg/m^2^ of municipal compost (a mixture of yard waste, food waste, and other municipal organics waste). These compost inputs were associated with single-season research projects, and after each growing season, soil from the raised bed garden plots was homogenized and redistributed. As a result, the long-term annual P input to these garden plots was approximately 15 g P/m^2^, 50% lower than the median compost-derived P input rate documented for urban gardeners in Minneapolis and Saint Paul [23]. Before the current experiment began in 2017, soil from all plots was again homogenized and redistributed. At the beginning of the current experiment, garden soil was high in organic matter (9.4%) and available P (Bray P 75 mg/kg) as a result of previous compost addition; similar to the median Bray P in urban gardens surveyed in Minneapolis and Saint Paul [17].

### Experimental design

The purpose of this study was to examine potential tradeoffs among management goals of maximizing crop yields and compost-derived nutrient assimilation, and minimizing nutrient loss from leachate. Although initial garden soil had a surplus of P, typical of urban gardens [16,17,26], we examined a range of options using compost to fulfill all or part of the estimated N demand of crops. The rate of compost application in this study was relatively low, with the highest compost application rate (20 g P/m^2^/y) used in this study being one-third lower than the median application rate (30 g P/m^2^/y) that we previously documented for local urban gardens [17].

The treatments represent different soil amendments to existing garden soils in the form of composts and/or synthetic fertilizer targeted to fulfill the estimated crop N and/or P demand. Each plot was randomly assigned one of six treatments: (1) a low level of municipal compost (mixture of food waste, yard waste, and other municipal organics waste) based on estimated crop P demand, supplemented with inorganic N fertilizer to meet estimated crop N demand (“Municipal P”); (2) a high level of municipal compost based on estimated crop N demand (“Municipal N”); (3) a low level of manure compost based on estimated crop P demand, supplemented with inorganic N fertilizer to meet estimated crop N demand (“Manure P”); (4) a high level of manure compost based on estimated crop N demand (“Manure N”); (5) inorganic N and P corresponding to estimated crop N and P demand (“Synthetic Fertilizer”); (6) a control treatment with no fertilizer application (“No Fertilizer”).

Treatments were randomly assigned to 32 raised bed plots, with each treatment replicated five times (Manure N, Manure P, Municipal N, Municipal P) or six times (Synthetic Fertilizer, No Fertilizer). Each 4 m^2^ plot is divided into four 1 m^2^ subplots, each with a different crop type. The crops used in this study are commonly used in UA and represent four different plant families: bell peppers (Solanaceae); bush beans (Fabacae); carrots (Apiacea); and cabbage (year 1) or collards (year 2) (Brassicaceae). The four crops are rotated annually in a clockwise direction within the plots. The subplots received ambient rainfall and supplemental irrigation (in equal amounts across plots) as needed depending on the antecedent rainfall conditions.

Crops were planted on June 1, 2017 and May 24, 2018. We used X3R Red Knight (F1) pepper seeds, E-Z Pick Organic bean seeds, Nectar Organic Pelleted (F1) carrot seed, Omero (F1) cabbage seed, and Flash collard seeds purchased from Johnny’s Selected Seeds. Peppers, beans, cabbages and collards were started in a greenhouse and transplanted after reaching a height of approximately 10 cm. Carrots were planted directly from seed. Peppers were planted in two rows of three (6 plants/m^2^), beans in two rows of six (12 plants/m^2^), cabbages/collards in two rows of three (6 plants/m^2^), and carrots in three rows of twenty (60 plants/m^2^). Any seedlings that died during the first two weeks of the experiment were replaced.

### Soil amendments based on crop N or P demand

We estimated the N, P, and K requirement for each of the crops based on anticipated yield expectation from the plots using the USDA yield calculator (see S1 Table). We determined compost application rates based on N or P as the priority nutrient dictated by the prescribed treatment. The amount of compost to apply as per the specific crop N or P demand was estimated using the bulk density and total N and P content measurements of the compost, based on measurements made from these same compost sources in the year before the current experiment began (2016). The rate of compost application for the different crops across the six treatments is detailed in the Supplement (see S2 Table). Since compost is a slow-release fertilizer, compost was applied at a rate assuming only 20% of the total N and 40% of the total P in the compost would be plant-available the first year after it is applied [27]. Compost application rates are therefore based on plant-available N and P rather than the total N and P, and they are the amount of N and P made available to plants over the growing season after fertilization (S2 Table). The synthetic fertilizer treatment applied commercially available N (26-0-2) and P (0-16-0) fertilizers corresponding to estimated crop N and P demand. When compost was applied based on anticipated crop P demand, additional synthetic N fertilizer (26-0-2) was added to meet estimated crop N demand, after accounting for the plant available N portion from the applied compost. When compost was applied based on anticipated crop N demand, P was necessarily added in excess of crop demand. Compost was applied to the soil at the start of each growing season (during the last week in May) and was mixed into the top 10 cm of garden soil. N fertilizer was applied on five dates each growing season at 14-day intervals.

The actual mass of N and P applied to the plots each year was calculated based on measured values of compost bulk density and N and P content. Bulk density was calculated by taking the weight of a known volume of sample after oven drying at 105°C overnight. At the start of each growing season, samples of the two composts were sent to the University of Minnesota Research Analytical lab for analysis of total C, N, P, and K content.

### Crop nutrient and biomass analysis

In 2017, peppers were harvested 11 times (between 08/04 to 10/18), beans 14 times (07/10 to 10/18), and carrots and cabbages once in mid-August. In 2018, peppers were harvested 12 times (between 07/12 to 10/29), beans 12 times (07/10 to 10/05), and carrots 2 times (08/09 and 09/26) and collards 14 times (07/06 to 10/29). At the end of the season, whole plant including roots were harvested and weighed for all crops. Fresh wet weight of the harvested biomass (including stems and leaves for carrots) was measured from each of the subplots. During one harvest in mid-August, a subsample of harvested biomass from each subplot was collected, weighed, dried at 60°C for 72 hours and dry weights were taken to calculate dry weight: wet weight ratios for each crop. The dried plant tissue subsamples were processed for C, N, and P content by grinding them into fine powder (<0.5 mm). Total C and N was determined by dry combustion where 250–350 mg of air-dried, pulverized sample was weighed into a capsule and combusted at 900⁰C in the presence of oxygen within a quartz combustion tube followed by subsequent measurement of CO_2_ and N_2_ evolution in a CN analyzer (Elementar, Inc. VarioMAX C/N Analyzer, Ronkonkoma, NY) [28]. Total P was analyzed by pre-digesting a 0.25 g sample of dried plant material for 60 minutes with 2 mL H_2_O_2_ and 0.5 mL HNO_3_. Sample was then digested in the microwave using modified Miller Digest method: 100°C for 8 minutes followed by 195°C for 12 minutes, and extract concentrations analyzed by Inductively Coupled Plasma Atomic Emission Spectrometry (ICP-AES) [29]. Growing season total harvestable fresh (i.e., wet weight) biomass was obtained by summing all the individual harvests’ biomass. The ratio of crop dry weight: wet weight was applied to estimate dry weights from corresponding fresh weights per subplot.

### Leachate nutrient flux

Before planting the crops, lysimeters were installed in the center of each 1 m^2^ subplot at 0.4 m depth for leachate water collection. Lysimeters were custom-designed [22] and comprised of a 23-cm diameter (0.0129 m^2^) and 11.8-cm diameter (0.0109 m^2^) plastic funnel in 2017 and 2018 respectively, secured to a 1 L wide-mouth plastic Nalgene bottle. Tygon tubing (inside diameter of 0.2 cm) extended from the base of the Nalgene bottle through the funnel, to 30 cm above the plot surface for leachate sampling. Rock wool was placed in the funnel around the tubing to ensure that the tubing is secured in place and avoid entry of any solid particles into the bottle. In the beginning of every field season, old lysimeters from all 128 subplots (and five extra locations from the adjacent grass lawn, serving as reference) were replaced with new ones.

Leachate water from lysimeter was collected weekly from May 30 to October 25, 2017 and May 29 to November 7, 2018 using a polypropylene syringe with attached 3-way stopcock. The total volume of leachate was measured, and a 20-mL leachate subsample was transferred in a plastic scintillation vial for analysis of PO_4_^3-^concentrations. Leachate concentrations were analyzed from those lysimeters for which >5 mL of leachate was recovered. Water samples were either run immediately, refrigerated if run within 48 hours, or frozen if run later. Leachate PO_4_^3-^ concentrations were analyzed using Hanna Instruments Phosphate Low Range Portable Photometer (HI96713). Leachate nutrient mass was calculated for individual sampling events (n = 23 in 2017, n = 24 in 2018) by multiplying nutrient concentration of the subsample by the total water volume: nutrient mass (mg) = nutrient concentration (mg L^-1^) × leachate volume (L). The resulting mass numbers were divided by the lysimeter funnel area to express results in per square meter of garden surface area. Cumulative mass of nutrients exported from the garden plots over the growing season was determined by summing all the individual mass flows:

$$Cumulative\;nutrient\;mass=\sum_{i=1}^{n}leachate\;oncentration\times leachate\;volume$$ (Eq 1)

where *n* equals the number of weekly sampling periods.

### Nutrient inputs, recovery and loss

The volume of compost P or N required for each of the subplot to meet the specific crop was calculated based on bulk density and total P or N content measurements of these two types of compost made prior to the start of the experiment, in 2016. Actual mass of N and P added from compost was calculated based on actual measurements of bulk density, and total P and N content, from each year's batch of compost (Table 1). A greater volume of the manure compost was required relative to the municipal compost to meet the same level of P or N demand of the crop. When compost was applied to meet the P demand, supplemental N was added as 26-0-2 synthetic fertilizer, after accounting for the supply of plant available N from the compost. By the nature of the calculations, when compost is applied to meet the P demand, there is variation in the N inputs between the two compost types, whereas when compost is applied to meet the N demand, P inputs vary between the compost types (See S2 Table). While the composts were added all at once in the beginning of the season, synthetic N fertilizer was added biweekly over a period of five events in the Synthetic Fertilizer and “P” treatments, and synthetic P was added once in the beginning in the Synthetic Fertilizer treatment.

**Table 1:** Manure and municipal compost physical and chemical properties averaged for 2017 & 2018.

Properties	Unit	Municipal Compost	Manure Compost
Bulk density	dry mass g L^-1^	366.7	97.7
Total C	%	22.5	38.0
Total N	%	1.4	0.8
Total P	%	0.2	0.2
Total K	%	0.7	0.5

Nutrient P recovery was calculated as the mass of nutrients removed from the plots via plant harvest. The total amount of nutrients recovered in plants was determined by multiplying tissue nutrient concentration of N or P by the total crop biomass harvested over the course of the growing season. Calculations were based on dry weights. Nutrient loss is defined as the total mass of nutrients transported or “lost” to soil pore water as leachate (Eq 1) from the upper soil layers to potentially the groundwater. The final nutrient mass balance calculations were estimated at the plot level (e.g., 4 m^2^) by summing the P inputs and outputs (e.g., plant harvest and losses via leachate) across the four subplots over the two years.

### Soil analysis

Soil samples were collected from the top 10 cm of the subplots, once in the beginning for the initial homogenized soil in June 1, 2017. Additionally, composite soil samples at the treatment level were also collected at the end of the growing season from one of the subplots (e.g., subplot “A”) on October 31, 2017 and November 5, 2018 and analyzed for organic matter, pH, NO_3_^-^, Bray-P and total P concentrations at the University of Minnesota Research Analytical Lab. Soil pH was determined on a 1:1 volumetric ratio of soil/water mixture composed of a 10 g NCR-13 volumetric soil scoop and 10 mL double-deionized water by Mettler Toledo Seven-Multi pH meter (Mettler-Toledo, LLC, Columbus, OH). For determination of organic matter content, a 5 g NCR-13 volumetric scoop of soil was dried for 2 h at 105°C and weighed. The sample was ashed for 2 h at 360°C and reweighed. The resulting loss of weight, as a percentage of the dry soil, was estimated as the organic matter content. NO_3_^-^ was extracted by shaking 2 g of air-dried soil in 30 mL 0.01 M CaSO_4_ for 15 minutes followed by filtration and concentrations were analyzed on a Lachat Quikchem 8500 Flow Injection Analyzer (Lachat Instruments, Loveland, CO). Bray-P was extracted by shaking 1 g of air-dried soil in 10 mL of 0.025 M HCl and 0.03 M NH_4_F for 5 minutes and P concentrations determined on the filtrate by the molybdate-blue method using ascorbic acid as a reductant on a Brinkmann PC 900 probe colorimeter. Total P was determined by digesting 0.5 g of air-dried soil with 10 mL of HNO_3_ in a 50 mL quartz vessel using microwave digestion for 6.5 minutes at 175°C and concentrations analyzed by inductively coupled plasma atomic emission spectrometry (ICP‐AES) [29]. Soil from the adjacent grass lawn was also concurrently measured in 2017 as reference.

### Plot maintenance

Plots were watered periodically based on recent precipitation history. Water was distributed evenly over each 4 m^2^ plot for a set amount of time (typically 30, 45, or 60 seconds, depending on soil moisture), and volume of water added was estimated by measuring the time required to fill an 11-L bucket at every watering event. Plots were maintained throughout the growing season by hand removal of weeds, approximately once per week. Japanese Beetles (*Popillia japonica*), which are considered pests, were removed by hand when encountered.

### Statistical analysis

Differences in crop yield (wet mass) and PO_4_^3-^ leachate across treatments were assessed using a three-way analysis of variance, in which crop wet mass was modeled as a function of year, crop, treatment, and treatment*year interaction. The crop yield and PO_4_^3-^ leachate variables were log-transformed to meet assumptions of normality. The statistical analyses were performed using JMP Pro 14.0.0 (SAS Institute Inc., Cary, NC, 2015). All results are reported as mean with standard error. A criterion of 95% confidence (α = 0.05) was used.

## Results

### Crop yield wet mass

**Fig 2:**
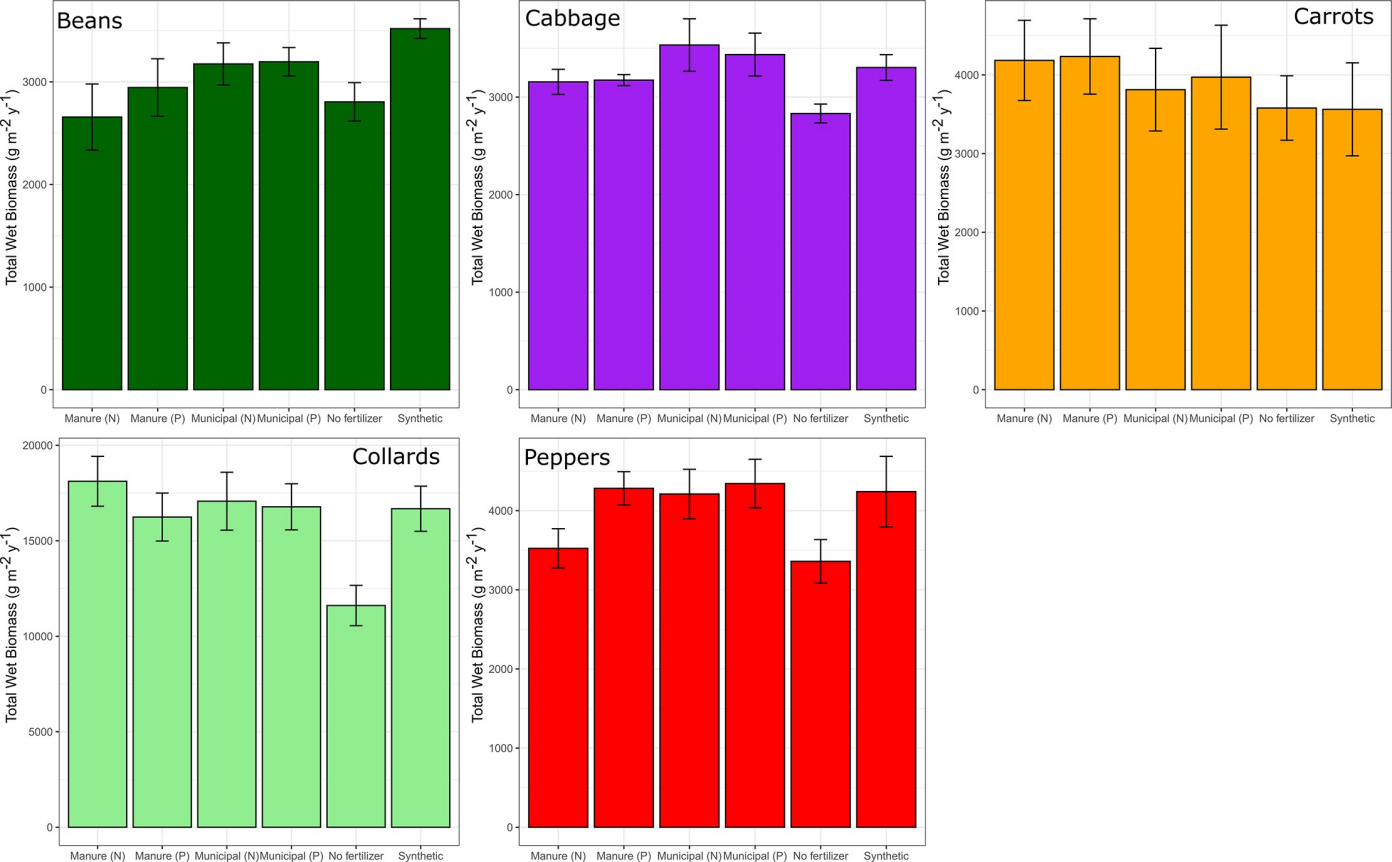
Mean total crop yield wet biomass (g m^-2^ y^-1^) combined for years 2017 and 2018 across the different treatments for each crop type. Note differences in scales for each crop.

There was a significant difference in log(yield) across crops (collard greens had the highest yield with a mean of 15,962 g/m^2^/y, and cabbage had the lowest yield with a mean of 3228 g/m^2^/y) and years (mean yields were higher in 2017 compared to 2018, despite the substitution of collards for cabbage). There was no significant overall treatment effect or treatment*year interaction (Table 2). However, treatments receiving soil amendments had approximately 26% greater crop yield relative to the No Fertilizer control treatment (Fig 2). The No Fertilizer control treatment had a mean yield of 6024 g/m^2^ (averaged across all crops) in 2017 and a mean yield of 4246 g/m^2^ in 2018. In 2017, mean yields (averaged across all crops) for the five treatments that received compost or fertilizer inputs ranged from 6373–6780 g/m^2^, compared to 5642–6067 g/m^2^ in 2018.

**Table 2:** Results of 3-way analysis of variance modeling log(yield) as a function of treatment, crop, year, and treatment*year interaction. R^2^ = 0.69.

	DF	Sum of Squares	F Ratio	P-value
Year	1	0.90	33.7	<0.0001
Treatment	5	0.22	1.6	0.1577
Crop	4	13.59	127.2	<0.0001
Year*treatment	5	0.06	0.4	0.8265

### Nutrients recycled from compost

**Fig 3:**
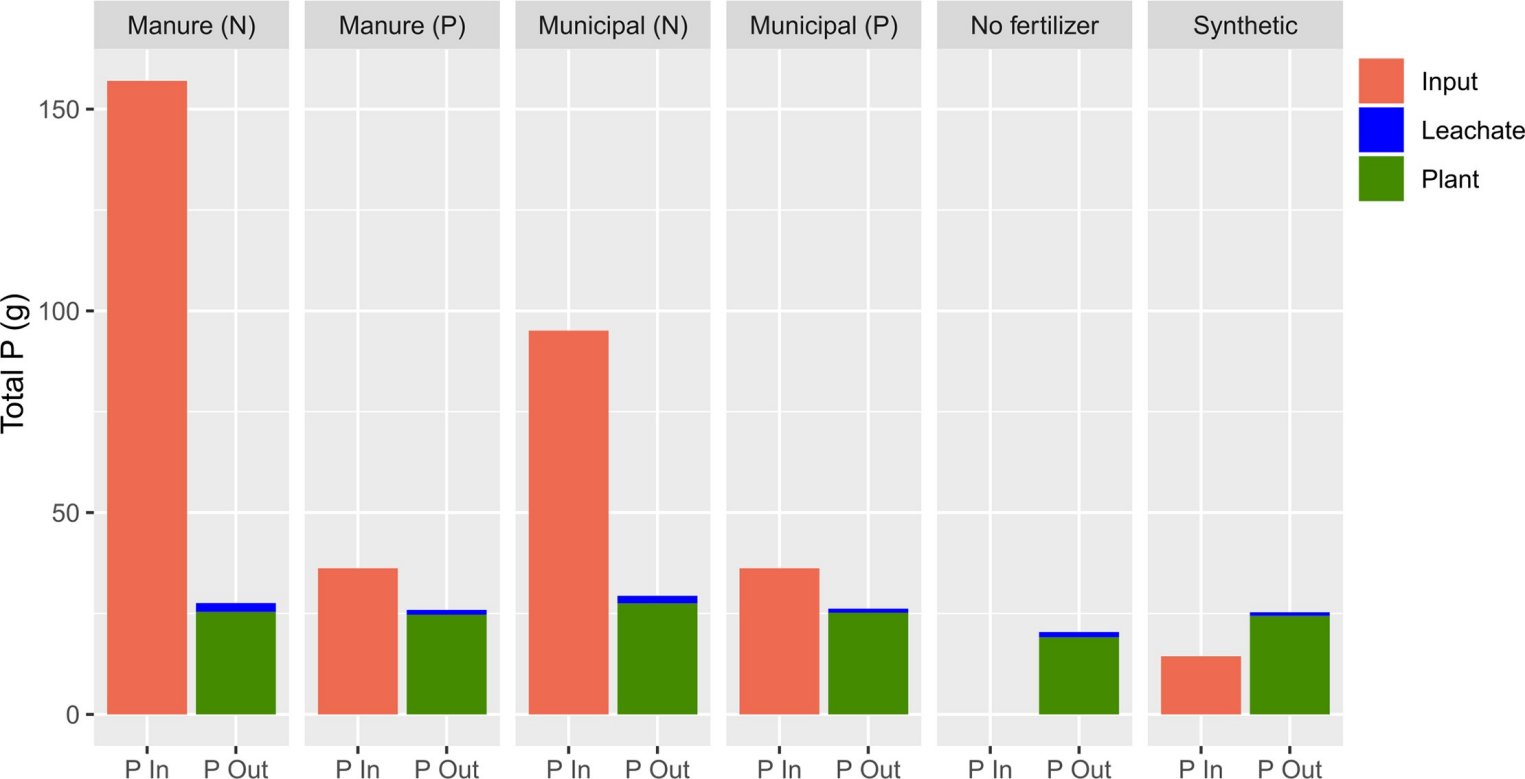
Mass balance at plot level (of 4 m^2^) showing mean P inputs, mean P export via plant harvest, and loss via dissolved inorganic P in leachate.

Total P applied as compost to the four compost treatments ranged from 36.2–157.0 g P/4m^2^/2y (Table 3). The mass of P recovered by crops ranged from 19.1 g P/4m^2^/2y (for the No Fertilizer Treatment) to 27.5 g P/4m^2^/2y (for the Municipal N treatment). The percentage of compost P inputs accounted for by recovered P in crops was 68% for the Manure P treatment and 69% for the Municipal P treatment (both of which were targeted to anticipated crop P demand), and was 16% for the Manure N treatment and 29% for the Municipal N treatment (Fig 3). The mass of P recovered by crops receiving compost in excess of control treatment means ranged from 5.6–8.4 g P/4m^2^/2y.

**Table 3:** Mean mass of P applied to 4m^2^ plot over two years, P recovered from crop yield, and P recovered from crops in excess of control treatment mean.

Treatment	Total P applied (g P/4m^2^/2y)	Crop Yield (g P/4m^2^/2y)		Excess Crop P (g P/4m^2^/2y)	P Leachate (g P/4m^2^/2y)	
	Mean	Mean	SE	Mean	Mean	SE
No fertilizer	0.0	19.1	0.7	—	1.3	0.3
Synthetic	14.4	24.4	0.7	5.3	0.9	0.1
Manure N	157.0	25.4	1.2	6.3	2.2	0.0
Manure P	36.2	24.7	1.4	5.6	1.2	0.3
Municipal N	95.1	27.5	1.9	8.4	1.9	0.5
Municipal P	36.2	25.2	1.8	6.1	1.0	0.2

### Leachate P

Log(leachate P) varied across crops (P = 0.0341), with collards having a significantly lower leachate flux compared to other treatments, and across years, with higher leachate PO_4_^3^ rates in 2018 (P<0.0001; Table 4). Treatment (P = 0.1456) and treatment*year interaction (P = 0.6604) were not significant factors in the model (Table 4).

**Table 4:** Results of 3-way analysis of variance modeling log(leachate P) as a function of treatment, crop, year, and treatment*year interaction. R^2^ = 0.21.

	DF	Sum of Squares	F Ratio	P-value
Year	1	22.84	49.6	<0.001
Treatment	5	3.82	1.7	0.1456
Crop	4	4.88	2.7	0.0341
Year*treatment	5	1.50	0.7	0.6604

**Fig 4:**
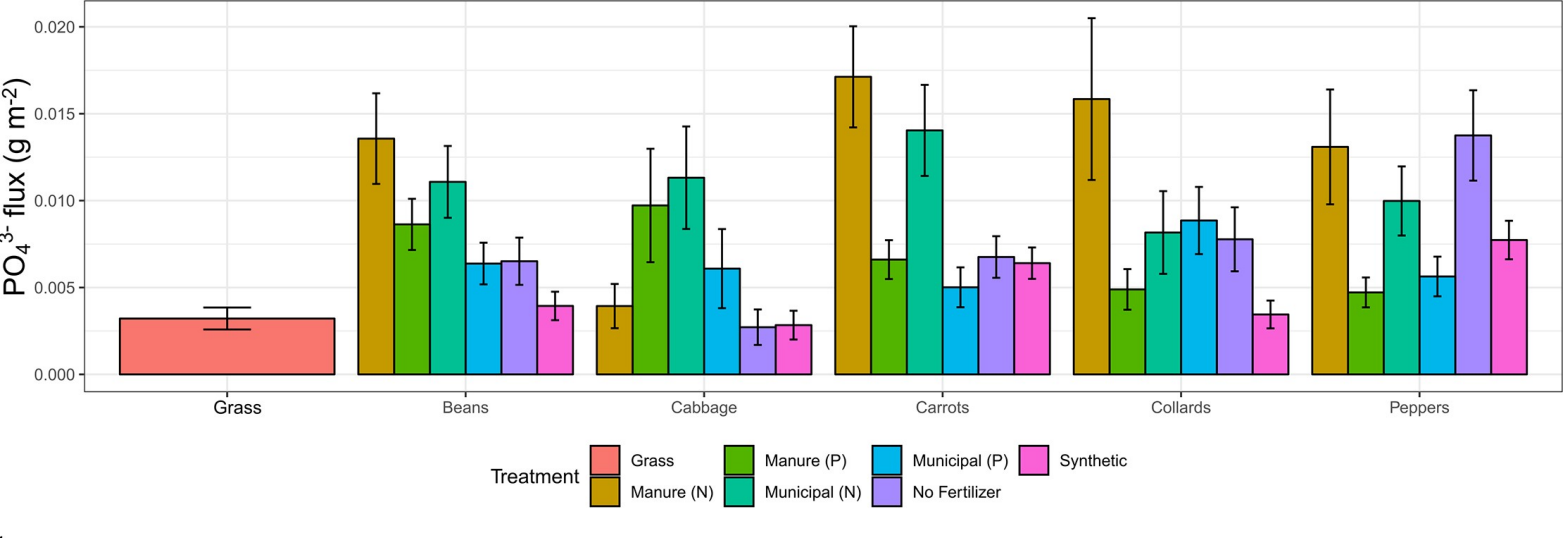
Mean leachate P fluxes ± 1 standard error across soil treatments per crop type. Leachate P flux from grass plots outside the garden is also included for reference.

Across subplots and across both years, Manure N and Municipal N had the highest mean leachate PO_4_^3-^ fluxes (Table 3). Leachate PO_4_^3-^ fluxes ranged from 0.7% of compost P added (for Manure P treatment) to 2.8% of compost P added (for Municipal P treatment; Table 2). Leachate PO_4_^3-^ fluxes were 2–4 times greater compared to reference grass plots (Fig 4).

### Soil nutrients

Soil OM content in all treatments except the No Fertilizer treatment increased relative to the initial condition. Bray-P concentrations of the initial garden soil was almost three times higher relative to the reference grass in 2017 (75 ppm vs. 26 ppm respectively). Bray-P concentrations increased in some of the treatments–especially the Municipal compost treatments–relative to the No Fertilizer control, which saw a decrease over the two years (Table 5). Total P concentrations also showed the same pattern in general, decreasing in the No Fertilizer control, while increasing in the Municipal compost treatments by the second year. Soil NO_3_^-^ concentrations in 2018 declined in most of the treatments, but particularly in the No Fertilizer treatment (Table 5), potentially indicating eventual N limitation. No Fertilizer treatment also had the lowest soil NH_4_^+^ concentrations among all the treatments in 2018 (Table 5).

**Table 5:** Soil chemical properties measured for initial homogenized soil (June 2017), and for different treatments at end of Year 1 (October 2017), and end of Year 2 (November 2018).

Treatment	OM (%)	pH	K (ppm)	NO_3_^-^ (ppm)	NH_4_^+^ (ppm)	Bray-P (ppm)	TP (ppm)
June 1, 2017							
Initial garden soil	9.4	7.1	74.5	7.4		75	701
October 31, 2017							
*Grass	5.1	7.2	165.2	7.1		26	
Manure (N)	8.6	7.0	118.6	7.7		74	729
Manure (P)	8.9	7.0	93.0	16.0		78	845
Municipal (N)	9.3	7.0	201.7	10.0		91	890
Municipal (P)	8.1	7.0	109.4	9.0		80	752
Synthetic	8.4	6.9	81.3	12.7		86	848
No fertilizer	8.2	7.0	76.0	9.8		74	831
November 5, 2018							
Manure (N)	10.5	7.1	134	9.9	17.2	71	745
Manure (P)	10.5	7.1	55	4.9	13.8	55	637
Municipal (N)	13.7	7.3	340	7.2	11.6	103	921
Municipal (P)	9.6	7.2	182.5	8.3	12.9	81	814
Synthetic	9.6	6.7	60	6.4	11.6	76	736
No fertilizer	8	7.2	89	3.8	8.4	65	676

*Grass included for reference.

## Discussion

Our results generally support the hypothesis that there are inherent tradeoffs in nutrient recycling in UA. Treatments that received the highest compost P inputs (and thus assimilated the most recycled P) had lower fraction of P recovered in crops relative to P inputs compared to other treatments, indicative of P buildup in the soil or loss through leachate. Although compost treatments did not show statistically significant differences in leachate P, treatments receiving higher levels of compost P had mean leachate P fluxes nearly double those observed in treatments receiving lower compost inputs and the No Fertilizer control treatment. Additionally, the Manure P treatment showed a 30% increase in soil Bray-P after two growing seasons, relative to initial conditions. While previous studies have indicated that UA cannot assimilate the mass of P in organic waste generated by cities [11], our study is the first to quantify the tradeoffs that constrain the potential of UA to recycle P from urban organic waste back into the human food system.

At the same time, our results also demonstrate that nutrient pollution, a potential ecosystem disservice from UA, can be minimized by targeting compost inputs based on anticipated crop nutrient demand. All treatments receiving compost had similarly high yields through the first two years of this study. Crop yields in treatments receiving compost were comparable to values reported from other observational [11,30,31] and experimental [22,32] studies despite compost input rates that were much lower than those typical of UA [11,15,16]. Likewise, leachate P rates measured in this study were approximately an order of magnitude lower than rates we documented in a previous study that used much higher compost input rates, typical of local UA practitioners [22].

While nutrient recycling has been recognized as an important potential benefit of UA [6,11,33], our results highlight the difficulty in quantifying this ecosystem service. Compost-bound nutrients could be considered “recycled” as soon as they are applied to an urban garden, in which case higher compost application would necessarily maximize nutrient recycling. In our study, applying compost based on anticipated crop N demand results in application rates 3–4 times higher than applying compost based on crop P demand (Table 3). However, crop yield P only accounted for 16% (Manure N treatment) and 28% (Municipal N treatment) of total P inputs, indicating that most of the P we added is accumulating in the soil. This metric of P-use efficiency (yield relative to inputs), over a long (e.g., decadal) time span, would be an appropriate metric of recycling, but since soil Bray-P was already high at the start of this study, and yields were relatively high in the No Fertilizer control treatment, it is likely that much of the P uptake by crops during the first two years of the study came from the existing pool of available P. A more conservative metric of P recycling would be quantifying crop yield P in excess of the control treatment. These values are less than 10% of the magnitude of compost P inputs (Table 3).

While the low N:P ratio that often characterizes urban compost poses challenges to recycling P (a non-limiting nutrient in many garden soils), our results show that input rates of P can be reduced by targeting compost inputs to crop P demand, and supplementing with synthetic N fertilizer to meet crop N demand. Other alternatives include using high-N soil amendments such as liquid fish emulsion, which has up to 6% N and 1.7% P content [34,35]. Abbasi et al. (2004) used fish emulsion in a peat mix to grow radish and cucumber seedlings and showed that fish emulsion was not only a source of nutrients for plant growth, but also contained disease suppressive properties [35]. Other potential sources of N recovered from waste streams include ammonium sulfates produced from digestates or wastewater [36,37].

We observed relatively high variability in water volumes among lysimeters, which could have affected flux rates. Pan (zero-tension) lysimeters such as the simple design used in this study have been shown to work reasonably well under wet conditions, but are less representative of actual flux rate as soil dries [38]. Divergence could lead to underestimating leachate volume, and as a result, underestimating associated leachate nutrient fluxes.

Our study focuses on outdoor, soil-based UA, because it is the form of UA that is increasingly common in urban areas with relatively low population densities (where land is available), and because it has potential to recycle urban nutrients. Many other forms of UA are practiced [39], and some forms of intensive indoor production using hydroponics or aeroponics can achieve high yields per unit area with high water and nutrient use efficiency. However, these practices do not recycle urban nutrients, so even if they lead to increases in local food production, they rely on imports of nutrients in addition to having high rates of energy usage [40,41].

## Conclusions

UA has the potential to supply many ecosystem services and help cities become more self-sufficient in food production. However, one potential ecosystem disservice from UA stems from nutrient loss due to the overapplication of compost, which could potentially contribute to impaired water quality. Our results illustrate the inherent tradeoffs between using UA as a sink for urban organic waste and optimizing nutrient recycling efficiency while minimizing nutrient loss through leachate. However, our results also show that careful compost application targeted to crop nutrient demands in UA can maintain high yields and minimize nutrient losses to leachate. UA has the potential to help close the urban nutrient loop, but if UA is to be scaled up in order to maximize potential social, economic, and environmental benefits, it is especially important to carefully manage nutrients to avoid ecosystem disservices from nutrient pollution.

## Supporting information

S1 Table Estimated crop N and P demand. (DOCX)

S2 Table Compost application rate to fulfill crop N and/or P demand. (DOCX)
