# Supplementary material for: Quantifying nutrient recovery efficiency and loss from compost-based urban agriculture
Source: PLoS One. 2020 Apr 3;15(4):e0230996. doi: 10.1371/journal.pone.0230996 (PMC7122751; doi:10.1371/journal.pone.0230996)
Supplement: S1 Table — (DOCX) [file pone.0230996.s001.docx]

**S1 Table.** Estimated crop N and P demand

| **Crop** | **Estimated yield**  **(kg wet mass m^-2^)^a^** | **Estimated total plant biomass**  **(kg wet mass m^-2^)^b^** | **Estimated total plant biomass**  **(kg dry mass m^-2^)^c^** | **Estimated yield**  **(g N m^-2^)^d^** | **Estimated yield**  **(g P m^-2^)^e^** |
| --- | --- | --- | --- | --- | --- |
| Bell pepper | 2.0 | 4.1 | 0.3 | 11 | 0.8 |
| Bush bean | 4.1 | 8.2 | 0.6 | 21 | 3.1 |
| Carrot | 4.1 | 5.3 | 0.4 | 8 | 1.9 |
| Cabbage | 4.1 | 5.3 | 0.4 | 18 | 1.4 |

^a^ Estimated from vegetable yield calculator: <http://www.ufseeds.com/Crop-Calculators.html>; <https://plants.usda.gov/npk/main>

^b^ We assumed total biomass: harvest ratios of 2 for beans and peppers, and 1.3 for cabbage and carrots

^c^ We assumed average dry matter for all crops of 7.6% (Stanhill 1977)

^d^ Peppers: 0.27%N by wet mass, Neuwiler 2011; Green Beans: 0.26%N by wet mass, FAO 2011; Carrots: 0.14%N by wet mass, FAO 2011; Cabbage: 0.33%N by wet mass, Neuwiler 2011

^e^ Cabbage: 0.026%P by wet mass; carrots: 0.035%P by wet mass; peppers: 0.020% by wet mass; green beans: 0.038%P by wet mass (https://ndb.nal.usda.gov)
