# Supplementary material for: Quantifying nutrient recovery efficiency and loss from compost-based urban agriculture
Source: PLoS One. 2020 Apr 3;15(4):e0230996. doi: 10.1371/journal.pone.0230996 (PMC7122751; doi:10.1371/journal.pone.0230996)
Supplement: S2 Table — (DOCX) [file pone.0230996.s002.docx]

**S2 Table.** Compost application rate to fulfill crop N or P demand.

| **Crop** | **Treatment** | **Crop N requirement** | **Crop P requirement** | **Compost N required^a^** | **Compost P required^a^** | **Volume compost required^b^** | **^c^PAN as compost** | **Additional N required** |
| --- | --- | --- | --- | --- | --- | --- | --- | --- |
|  |  | **g N m^-2^** | **g P m^-2^** | **g N m^-2^** | **g P m^-2^** | **L** | **g N m^-2^** | **g N m^-2^** |
| Bell pepper | Municipal N | 11 |  | 55 |  | 10.2 |  |  |
| Bell pepper | Municipal P | 11 | 0.8 |  | 2 | 2.6 | 2.8 | 8.2 |
| Bell pepper | Manure N | 11 |  | 55 |  | 27.9 |  |  |
| Bell pepper | Manure P | 11 | 0.8 |  | 2 | 2.9 | 1.1 | 9.9 |
| Bell pepper | Synthetic | 11 | 0.8 |  |  | 0 |  |  |
| Bell pepper | No fertilizer |  |  |  |  | 0 |  |  |
| Bush bean | Municipal N | 21 |  | 105 |  | 19.4 |  |  |
| Bush bean | Municipal P | 21 | 3.1 |  | 7.8 | 9.9 | 10.6 | 10.3 |
| Bush bean | Manure N | 21 |  | 105 |  | 53.3 |  |  |
| Bush bean | Manure P | 21 | 3.1 |  | 7.8 | 11.2 | 4.4 | 16.6 |
| Bush bean | Synthetic | 21 | 3.1 |  |  | 0 |  |  |
| Bush bean | No fertilizer |  |  |  |  | 0 |  |  |
| Carrot | Municipal N | 8 |  | 40 |  | 7.4 |  |  |
| Carrot | Municipal P | 8 | 1.9 |  | 4.8 | 2.4 | 6.5 | 1.5 |
| Carrot | Manure N | 8 |  | 40 |  | 20.3 |  |  |
| Carrot | Manure P | 8 | 1.9 |  | 4.8 | 6.9 | 2.7 | 5.3 |
| Carrot | Synthetic | 8 | 1.9 |  |  | 0 |  |  |
| Carrot | No fertilizer |  |  |  |  | 0 |  |  |
| Cabbage | Municipal N | 18 |  | 90 |  | 16.7 |  |  |
| Cabbage | Municipal P | 18 | 1.4 |  | 3.5 | 4.5 | 4.8 | 13.2 |
| Cabbage | Manure N | 18 |  | 90 |  | 45.6 |  |  |
| Cabbage | Manure P | 18 | 1.4 |  | 3.5 | 5.0 | 2.0 | 16.0 |
| Cabbage | Synthetic | 18 | 1.4 |  |  | 0 |  |  |
| Cabbage | No fertilizer |  |  |  |  | 0 |  |  |

^a^ Since compost is like a slow-release fertilizer, we assume only 20% of the N and 40% of the P in the compost will be plant-available the first year after it is applied. ^b^ Manure compost (based on 2016 data): bulk density 116 g dm/L, 1.7%N by dm, 0.60%P by dm; Municipal compost (based on 2016 data): bulk density 491 g dm/L, 1.1%N by dm, 0.16%P by dm. ^c^Plant Available N (PAN) is the amount of N made available to plants over the growing season after fertilization.
